# Supplementary material for: Male sterile 305 Mutation Leads the Misregulation of Anther Cuticle Formation by Disrupting Lipid Metabolism in Maize
Source: Int J Mol Sci. 2020 Apr 3;21(7):2500. doi: 10.3390/ijms21072500 (PMC7177535; doi:10.3390/ijms21072500)
Supplement: Supplementary file 1 [file ijms-21-02500-s001.zip › Supplementary File/Table S1.docx]

**Table S1.** The primers for qPCR analysis used in this study.

| **Unigene ID** | **Gene description** | **Sequence (5′-3′)** |
| --- | --- | --- |
| GRMZM2G143625 | uncharacterized LOC100284973 | F: GACTACTACGTGTGCCTGGT |
|  |  | R: GCACAGGTAGAGATAGCGGT |
| GRMZM2G072205 | 3-oxoacyl-[acyl-carrier-protein] synthase II chloroplastic | F: ACCATACCTGCCCATTGTCA  R: TAAGATCCCATCACCTCCGC |
| GRMZM2G117064 | long chain acyl-CoA synthetase 9 | F: GGCTTGCAAAGACCTGTTGA  R: CCTCAGCTGCCAATTCAAGG |
| GRMZM2G151087 | PAS2 | F: ACTGGGTCGTCTTCTTCGGA  R: CATGGAAGACTTGCTAGTG |
| GRMZM2G013082 | uncharacterized LOC100501739 | F: GCTCGCAGTCTCGCACAT  R: ATGGCAACGAAGATGGA |
| GRMZM2G075140 | 3-ketoacyl-CoA synthase 2 | F: GTCAACCACTACAAGCTCCG |
|  |  | R: GGAAGAGGCAGTTGGACATG |
| GRMZM2G126010 | Actin1 | F: AGAACTGCGACTGCCTCCAAAG  R: AGATGAGCAGGGTGCCCATTC |
