# Supplementary material for: Male sterile 305 Mutation Leads the Misregulation of Anther Cuticle Formation by Disrupting Lipid Metabolism in Maize
Source: Int J Mol Sci. 2020 Apr 3;21(7):2500. doi: 10.3390/ijms21072500 (PMC7177535; doi:10.3390/ijms21072500)
Supplement: Supplementary file 1 [file ijms-21-02500-s001.zip › Supplementary File/Table S2.docx]

**Table S2.** Fold change and annotations of common DEGs at different development stages.

| **Unigene ID** | **Gene annotation or description** | **Relative expression (Log2 ratio)** | | |
| --- | --- | --- | --- | --- |
|  |  | **FA/MA** | **FB/MB** | **FC/MC** |
| GRMZM2G117971 | Uncharacterized protein | 6.77 | 6.18 | 4.5 |
| GRMZM2G087824 | Uncharacterized protein | 2.1 | 2.3 | 3.23 |
| GRMZM2G097297 | O-methyltransferase ZRP4-like | 2.95 | 1.45 | 4.02 |
| GRMZM2G028393 | Protease inhibitor | 1.31 | 3.46 | 2.85 |
| GRMZM2G103748 | Unknown mRNA | 2.35 | 3.02 | 3.71 |
| GRMZM2G340656 | Alkaline alpha galactosidase 1 | 2.25 | 3.23 | 5.85 |
| GRMZM2G026980 | Xyloglucan endotransglucosylase | 5.8 | 4.21 | 6.27 |
| GRMZM2G104204 | Homeobox-leucine zipper protein | 2.42 | 2.42 | 2.88 |
| GRMZM2G078472 | Asparagine synthetase | 5.7 | 3.03 | 3.32 |
| GRMZM2G026143 | Uncharacterized protein | 2.43 | 1.82 | 2.34 |
| GRMZM2G099984 | Hydrophobic protein LTI6B | 1.91 | 1.61 | 2.35 |
| GRMZM2G047299 | Brain protein 44-like protein | 1.2 | 1.2 | 2.8 |
| GRMZM2G084935 | Polcalcin Jun o 2 | 1.14 | 1.03 | 1.18 |
| GRMZM2G133718 | Serine carboxypeptidase | 2.45 | -1.21 | -2.74 |
| GRMZM2G001205 | ZFP16-1 | 4.29 | 6.47 | 4.99 |
| GRMZM2G174719 | Adhesive/proline-rich protein | 3.12 | 2.21 | 3.16 |
| GRMZM2G374971 | Zeamatin | 1.99 | 1.44 | 3.54 |
| GRMZM2G075456 | Calcium binding EF-hand protein | 2.18 | 3.19 | 2.03 |
| GRMZM2G094510 | Uncharacterized protein | 1.52 | 1.27 | 3.01 |
| GRMZM2G164229 | Unknown mRNA | 3.92 | 1.36 | 3.72 |
| GRMZM2G005624 | Uncharacterized protein | 1.6 | 2.27 | 3.07 |
| GRMZM2G053206 | Uncharacterized protein | 4.25 | 5.55 | 5.17 |
| GRMZM2G014055 | Thioredoxin H-type | 1.84 | 1.98 | 4.19 |
| GRMZM2G132238 | Putative metacaspase family protein | 1.23 | 1.68 | 2.07 |
| GRMZM2G090245 | Aldehyde dehydrogenase | 4.2 | 8.37 | 7.84 |
| GRMZM2G456217 | Vignain (peptidase activity) | 7.05 | 4.32 | 1.39 |
| GRMZM2G354909 | Neomenthol dehydrogenase | 2.31 | 2.78 | 4.28 |
| GRMZM2G117942 | Barwin-like | 6.89 | 7.02 | 4.97 |
| GRMZM2G471357 | Peroxidase | 5.09 | 5.88 | 7.37 |
| GRMZM2G016435 | Dynein light chain LC6 | 3.14 | 3.67 | 3.74 |
| GRMZM2G397261 | Uncharacterized protein | 4.05 | 4.49 | 3.97 |
| GRMZM2G141322 | Ribonuclease 1 | 5.96 | 2.78 | 2.22 |
| GRMZM2G120079 | Putative metacaspase family protein | 1.85 | 1.94 | 3.84 |
| GRMZM2G156632 | Proteinase inhibitor WIP1 | 3.86 | 6.78 | 5.65 |

F, fertile sibling; M, ms305. A, B, and C represent pollen mother cell, dyads, and tetrad stages, respectively.
