# Supplementary figures and images for: Male sterile 305 Mutation Leads the Misregulation of Anther Cuticle Formation by Disrupting Lipid Metabolism in Maize
Source: Int J Mol Sci. 2020 Apr 3;21(7):2500. doi: 10.3390/ijms21072500 (PMC7177535; doi:10.3390/ijms21072500)

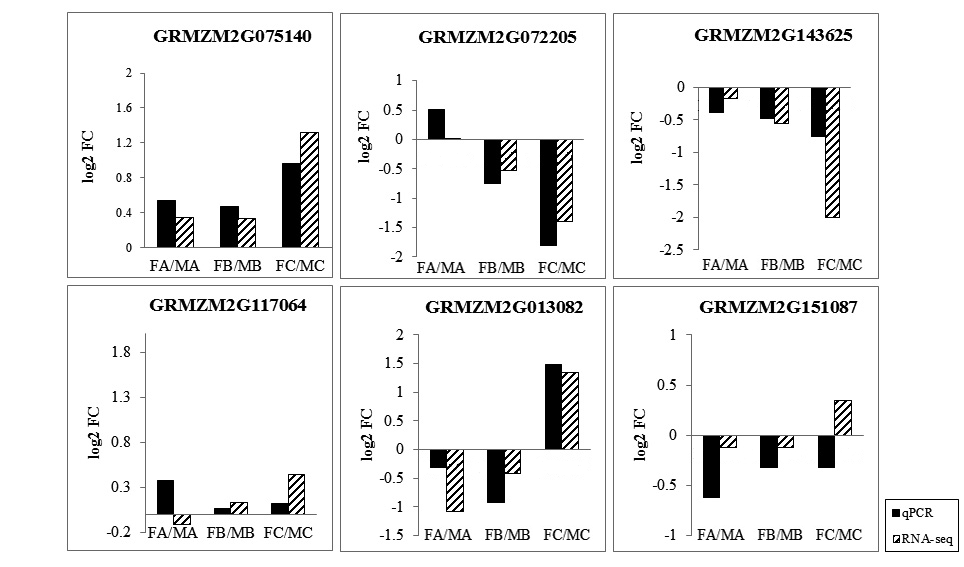

Supplement: Supplementary file 1 [file ijms-21-02500-s001.zip › Supplementary File/Figure S1.png]

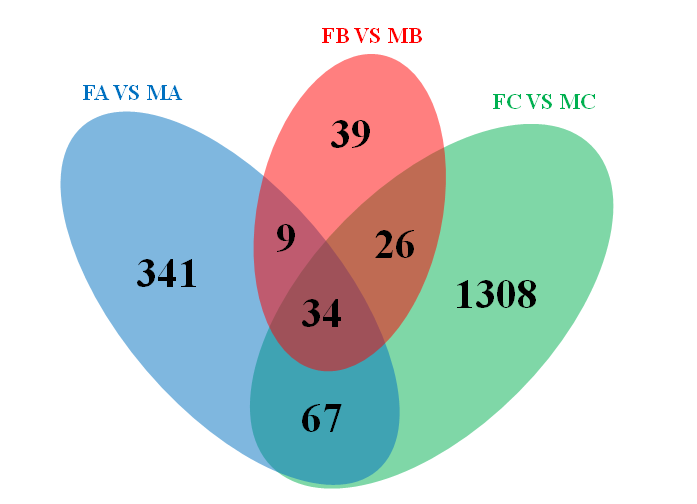

Supplement: Supplementary file 1 [file ijms-21-02500-s001.zip › Supplementary File/Figure S2.png]

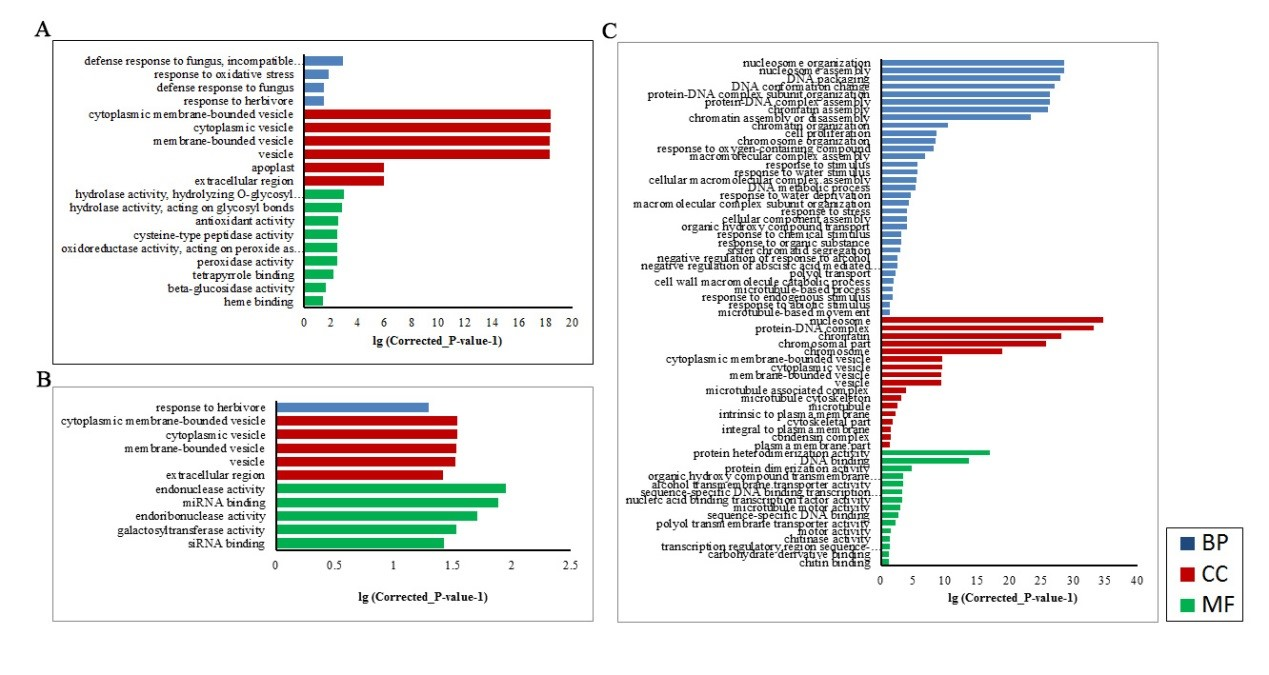

Supplement: Supplementary file 1 [file ijms-21-02500-s001.zip › Supplementary File/Figure S3.png]
